# Emerging Speech-in-Noise Tools for the Assessment of Hearing Loss: A Scoping Review

**DOI:** 10.3390/audiolres16020057

**Published:** 2026-04-11

**Authors:** Andrea Migliorelli, Marianna Manuelli, Chiara Visentin, Chiara Bianchini, Francesco Stomeo, Stefano Pelucchi, Nicola Prodi, Andrea Ciorba

**Affiliations:** 1 ENT & Audiology Unit, Department of Neurosciences, University Hospital of Ferrara, 44100 Ferrara, Italy; 2 Department of Engineering, University of Ferrara, Via Saragat 1, 44122 Ferrara, Italy

**Keywords:** speech-in-noise, hearing loss, digits-in-noise, hearing assessment, audiological screening

## Abstract

**Background/Objectives**: The objective of this scoping review was to map and critically describe emerging speech-in-noise assessment tools developed over the last decade for the evaluation of hearing loss beyond conventional audiological measures. **Methods**: This review was conducted in accordance with the Preferred Reporting Items for Systematic Reviews and Meta-Analyses extension for Scoping Reviews (PRISMA-ScR) guidelines. A comprehensive literature search was performed in the PubMed/MEDLINE, Scopus, and Embase databases. A comprehensive review of studies describing novel or emerging SIN-based assessment tools was conducted, with a particular emphasis on those including adult participants with normal hearing and hearing loss. **Results**: Nine studies met the inclusion criteria and were included in the review. The identified tools cover a range of methodological innovations, including advanced digits-in-noise paradigms, antiphasic and binaural presentation modes, optimized adaptive procedures, and digital or automated testing platforms. Several studies also incorporated artificial intelligence-based approaches, such as machine learning, text-to-speech, and automatic speech recognition, to enhance test development, administration, and hearing loss classification. Across all studies, SIN measures demonstrated the ability to reliably differentiate between normal hearing listeners and individuals with hearing loss and to provide complementary information beyond pure-tone audiometry. **Conclusions**: Emerging speech-in-noise tools show considerable potential to improve the functional assessment of hearing loss and to support more sensitive, accessible, and scalable approaches for hearing evaluation. Further research is required to assess their clinical integration and long-term impact on hearing screening and diagnostic pathways.

## 1. Introduction

Hearing loss is one of the most prevalent health conditions worldwide and represents a growing public health challenge [1]. Recent epidemiological evidence suggests that the prevalence of hearing loss is expected to increase substantially in the coming decades, with significant social, cognitive, and economic consequences [1]. Untreated hearing loss has been consistently associated with social isolation, reduced quality of life, cognitive decline, and an increased risk of dementia [2,3,4]. Despite these findings, the early identification of hearing loss in adults remains limited, with an average delay of several years between the onset of symptoms and the initiation of appropriate treatment [1].

Pure-tone audiometry (PTA) is widely accepted as the primary reference standard for clinical hearing assessment [5,6]. However, it is increasingly recognized that audiometric thresholds alone do not fully explain the listening difficulties reported by individuals in everyday life. In particular, many individuals experience considerable difficulties in understanding speech in noisy environments despite having normal or only mildly elevated pure-tone thresholds [5,7]. Furthermore, PTA necessitates quiet testing conditions, calibrated equipment, and specialized personnel, which restricts its applicability as a large-scale screening tool or for use in non-clinical settings.

In this context, the assessment of speech understanding in noise (speech-in-noise, SIN) is playing an increasingly crucial role in modern audiology. SIN tests aim to evaluate an individual’s functional ability to understand speech signals in the presence of background noise, a condition that more closely reflects real-world listening situations [8,9]. The most frequently used outcome measure is the speech reception threshold (SRT), defined as the signal-to-noise ratio (SNR) at which a listener correctly identifies a predefined proportion of the presented stimuli. Typically, the SNR is adjusted to achieve a 50% correct response rate in order to determine the SRT in noise [8,9,10,11]. SRT estimation is generally performed using adaptive procedures that adjust the SNR based on the listener’s responses, enabling an efficient and reliable assessment of auditory performance.

In recent years, the rapid methodological and technological advancement of speech-in-noise assessment has been facilitated by the presence of digital platforms, automated testing procedures, and novel stimulus presentation paradigms. Within a relatively short time frame, numerous innovative tools have been proposed, with the potential to reshape current strategies for hearing loss assessment.

In addition to their clinical utility, the development of online and self-administered SIN tests has been increasingly motivated by the need to improve the global awareness of hearing difficulties, particularly among older adults. Despite its high prevalence and well-documented impact, hearing loss often remains under-recognized, especially in its early stages, when impairments may primarily affect complex listening situations such as speech perception in noise rather than pure-tone thresholds [5,7]. This lack of awareness may contribute to delayed help-seeking and underdiagnosis, with potential consequences for communication, social participation, and cognitive health, as hearing loss has been consistently associated with cognitive decline and dementia [2,3,4,8]. In this context, easily accessible online SIN tests represent a valuable tool not only for screening purposes but also for increasing overall awareness, enabling individuals to self-assess their hearing abilities in ecologically relevant conditions. By reducing barriers to access and providing immediate feedback, these tools may facilitate the earlier recognition of hearing difficulties and promote timely referral for comprehensive audiological evaluation.

Considering these developments, there is a clear need to examine the new methodologies proposed for speech-in-noise assessment, focusing on the developed tools, on the methodological innovations introduced, and on the enhanced identification and characterization of hearing loss compared with traditional audiological measures.

The aim of this scoping review is to map and critically describe new tools for comprehensive speech-in-noise testing developed over the last ten years, with particular attention to their potential role in assessing individual hearing difficulties and in facilitating the identification of hearing loss.

## 2. Materials and Methods

**Figure 1:**
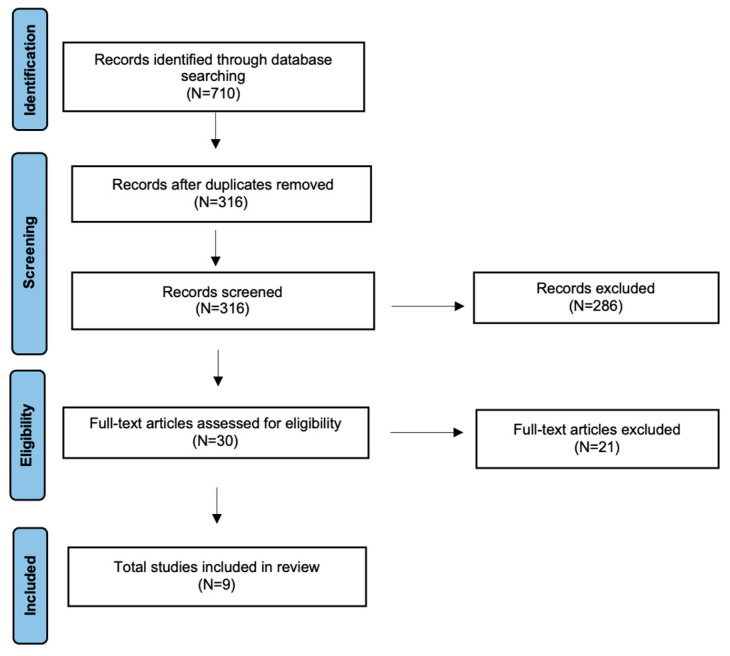
The literature review performed using PRISMA guidelines for scoping review.

This study was conducted as a scoping review following the Preferred Reporting Items for Systematic Reviews and Meta-Analyses extension for Scoping Reviews (PRISMA-ScR) guidelines [12]. The study selection process is shown in Figure 1. A comprehensive search of the English-language literature was performed using the PubMed/MEDLINE, Scopus, and Embase databases. These databases were selected for their broad coverage of the biomedical literature, while other resources such as Cochrane Library and UpToDate were not included as they primarily provide evidence syntheses and clinical summaries rather than comprehensive coverage of original research studies. The search covered publications from the last 10 years and combined controlled vocabulary terms and free-text keywords related to speech perception in noise, hearing assessment tools, and hearing loss. Search terms included combinations of “speech in noise”, “speech-in-noise”, “speech perception in noise”, “digits-in-noise”, “speech hearing test”, “hearing screening”, “hearing assessment”, “hearing loss”, and “listening difficulties”, together with terms such as “novel”, “new”, “emerging”, “digital”, and “automated”. Inclusion criteria were: (i) original research articles published within the last 10 years; (ii) studies describing speech-in-noise-based assessment tools or procedures; (iii) studies including adult populations with both normal hearing and hearing loss groups; (iv) studies proposing novel or emerging tools, paradigms, or analytical approaches aimed at identifying or characterizing hearing loss; (v) articles published in the English language. Exclusion criteria were: (i) studies focusing exclusively on speech perception in quiet; (ii) studies not involving speech-based auditory assessment; (iii) studies addressing hearing aid fitting, rehabilitation, or device optimization without comparison between participants with normal hearing and those with hearing loss; (iv) reviews, editorials, conference abstracts, or non-peer-reviewed articles; (v) studies published in languages other than English. Study selection was performed independently by two reviewers (AM and MM). Disagreements were resolved by discussion, and when necessary, by consultation with a senior reviewer (AC). The database search yielded 710 records. After removal of 394 duplicates, 316 unique articles were screened based on title and abstract. Thirty articles were selected for full-text evaluation. Following full-text assessment, nine studies met all eligibility criteria and were included in the final scoping review [9,10,13,14,15,16,17,18,19]. The reduction from the initially screened articles to those selected for full-text evaluation was based on strict adherence to the predefined inclusion and exclusion criteria outlined above. The protocol for this review has been registered on the Open Science Framework (OSF) Registries with the following registration link: https://osf.io/m359d (last access on 20 February 2026). 

## 3. Results

Nine studies published within the last decade were included in this scoping review [9,10,13,14,15,16,17,18,19]. The main features of the included studies are summarized in Table 1. Overall, the studies covered a broad range of SIN assessment tools and were conducted across multiple countries, reflecting an international research effort toward improving the assessment of hearing loss beyond conventional audiological evaluation. All studies included both normal hearing (NH) and hearing loss (HL) adult listeners, with sample sizes ranging from small experimental cohorts to large population-based samples. Digits-in-noise (DIN)-based tests represented the most frequently investigated tool, while sentence-based SIN tests, digital speech screeners, and machine learning-assisted screening approaches were also represented. The testing context varied from controlled clinical and laboratory settings to fully remote and self-administered platforms.

The types of speech-in-noise tools and their methodological innovations are detailed in Table 2. Most studies focused on closed-set speech materials, particularly digit triplets, reflecting an emphasis on reducing linguistic and cognitive demands while enabling efficient test administration. Sentence-based materials were used in fewer studies, primarily within clinical assessment contexts.

Several methodological innovations were identified. Adaptive procedures were refined to improve efficiency and reduce listening effort, as exemplified by Bayesian approaches applied to matrix sentence tests. Binaural presentation paradigms, including antiphasic digits-in-noise testing, were introduced to enhance sensitivity to asymmetric and conductive forms of hearing loss. In parallel, digital and mobile platforms enabled remote and self-administered testing, expanding the potential reach of SIN assessment.

Artificial intelligence-based approaches represented an additional area of innovation. These included the automated development of speech-in-noise tests using text-to-speech and automatic speech recognition, as well as machine learning methods applied to multi-feature SIN data for hearing loss classification. Together, these approaches highlight a shift from traditional test construction and scoring toward more automated and scalable assessment frameworks.

The outcome measures and clinical relevance of the included studies are summarized in Table 3. The reported quantitative metrics (e.g., sensitivity, specificity, accuracy, and reliability) are generally derived from comparisons with conventional audiological reference standards, such as PTA or established SIN tests, depending on the study design.

Across the included studies, SIN outcomes were primarily expressed as SRT, percentage-correct scores, or classification performance metrics. Quantitative results consistently demonstrated significant differences between normal hearing participants and those with hearing loss.

Studies based on the DIN paradigm reported clear SRT separation between groups. In the antiphasic DIN validation by Ceccato et al. [13], ROC analysis yielded sensitivity and specificity values of approximately 0.96 and 0.93 for detecting hearing loss above 20 dB HL. Similarly, De Sousa et al. [14] reported classification accuracies of approximately 75–79% in distinguishing normal hearing from unilateral or bilateral hearing loss using combined diotic and antiphasic DIN procedures.

Machine learning-based approaches also demonstrated strong classification performances. Lenatti et al. [15] reported accuracy values up to 0.85, with a sensitivity and specificity around 0.86 and 0.85, respectively, when multivariate models were applied to SIN screening data.

Digital screening tools showed comparable diagnostic performances. For instance, the digital Speech Hearing Screener proposed by Banks et al. [17] achieved classification accuracies ranging from 81.6% to 83.7% depending on the PTA threshold used.

Recent developments also include automated approaches for generating speech-in-noise tests. Polspoel et al. [19] introduced the ALADDIN framework (Automatic Language-independent Development of the Digits-in-Noise test), which uses text-to-speech and automatic speech recognition technologies to automatically generate DIN materials while maintaining a high diagnostic performance, with a reported sensitivity of 100% and specificity of approximately 84% for detecting hearing loss.

Other studies focused on the development of language-specific digital screening tools. For instance, Viola et al. [18] developed and validated an Italian digits-in-noise test capable of independently assessing each ear and providing reliable SRT estimates consistent with established audiological thresholds, supporting its potential use for smartphone-based hearing screening and clinical assessment.

Similarly, Fatehifar et al. [9] proposed a fully automated DIN test based on text-to-speech and automatic speech recognition. Their results showed good agreement with conventional DIN testing (mean bias −1.3 ± 4.9 dB) and acceptable test–retest reliability, suggesting that AI-driven automated approaches may represent a feasible solution for remote and self-administered hearing assessment.

In addition to diagnostic accuracy, several studies provided further quantitative descriptors of auditory performance. Schmid et al. [10] reported psychometric slope estimates and reduced listening effort using a Bayesian adaptive matrix-sentence procedure, while Polspoel et al. [16] examined the effects of presentation level on SRT measurements in antiphasic DIN paradigms.

Overall, the findings summarized in Table 3 indicate that emerging speech-in-noise tools extend beyond traditional single-metric outcomes and offer complementary information relevant for the screening, assessment, and triage of hearing loss.

## 4. Discussion

This scoping review analyzed and synthesized the main SIN assessment tools developed over the last decade with the aim of improving the identification and characterization of hearing loss beyond conventional audiological measures. Overall, the included studies highlight the rapid methodological and technological progress in the field of SIN testing, with a clear shift toward tools that are more sensitive, accessible, and capable of capturing functional aspects of hearing that are not fully represented by PTA. These developments further highlight the advantages of SIN testing over PTA. As these measures rely on suprathreshold and relative measures, they are less sensitive to variations in ambient noise and can be administered even in the absence of strictly controlled acoustic environments [5]. Furthermore, the ecological validity of SIN tests is greater, as they capture one of the most common listening challenges encountered in daily life [19]. These characteristics make SIN tests particularly attractive not only for clinical assessment (and particularly for those wearing hearing aids or cochlear implants), but also for hearing screening and for the identification of auditory deficits that may not be readily detected by conventional audiometry (i.e., speech processing disorders) [15,17,19].

Over the years, a wide range of SIN tests have been developed using different types of speech material, including sentences, words, and digits [9]. Sentence-based tests, including matrix sentence tests, facilitate the precise estimation of the SRT due to the slope of the intelligibility psychometric function [11,20,21]. However, in certain instances, these tests may present a cognitive challenge to the listener. Digit-based tests, and in particular DIN tests, have emerged as one of the most widely adopted solutions owing to their simplicity, reliability, and reduced dependence on linguistic and cognitive factors [16,18,22]. Nowadays, DIN tests are commonly used both in clinical practice and as self-administered tools for hearing screening.

Despite the widespread adoption of SIN tests, several challenges remain related to their development, standardization, and implementation [9]. The creation of perceptually equivalent speech stimuli has historically involved the use of professional recordings and complex validation procedures. Conversely, many SIN tests were originally developed for controlled clinical environments, which may limit their suitability for large-scale deployment or remote administration. These limitations produced substantial research efforts in order to make speech-in-noise assessment more efficient, accessible, and sensitive to different configurations of hearing loss.

Beyond their widespread adoption, the present review highlights the pivotal role of DIN tests as a reference paradigm for the development of new SIN tools [9,13,14,16,18,19]. Most of the included studies employed digit-based materials, taking advantage of their simplicity, reduced dependence on linguistic and cognitive skills, and high reproducibility. These characteristics make DIN tests particularly suitable for hearing screening and functional assessment in heterogeneous populations, including individuals with mild hearing loss or listening difficulties that may not be immediately evident on pure-tone audiometry.

Within this context, several studies have introduced innovative methodological variants of the DIN paradigm, demonstrating that relatively simple modifications to stimulus presentation can substantially increase test sensitivity. In particular, the use of binaural antiphasic presentations demonstrated an enhanced ability to differentiate between normal hearing listeners and those with hearing loss, including cases of unilateral or conductive types [13,14,16]. These findings suggest that the antiphasic paradigm represents a meaningful evolution of the traditional DIN test, enabling not only hearing screening but also an initial form of functional triage based on the configuration of the hearing deficit.

A second area of innovation concerns the optimization of procedures for estimating the speech reception threshold in noise. A number of the included studies put forward the implementation of sophisticated adaptive methodologies, including Bayesian procedures applied to matrix sentence tests, with the aim of reducing test duration and listening effort without compromising estimation accuracy [10]. From a clinical perspective, these approaches are particularly relevant because they address one of the longstanding limitations of sentence-based SIN tests, namely the high cognitive load and potential listening fatigue experienced by participants, especially older adults or individuals with hearing loss.

Another significant finding of this review is the increasing integration of digital platforms and automated tools in speech-in-noise assessment [9,10,13,15,17,19]. Several studies demonstrated the feasibility and reliability of self-administered SIN tests delivered via smartphones or web-based platforms. These studies have shown that these tools can maintain a good discriminative ability between normal hearing listeners and those with hearing loss, even outside traditional clinical settings. This evolution has important implications for large-scale hearing screening, as it helps overcome many of the logistical barriers associated with conventional audiological assessment.

In this context, some studies also explored the use of text-to-speech (TTS) and automatic speech recognition (ASR) technologies for the creation and management of fully automated SIN tests [9]. The use of synthetic speech stimuli in combination with automated scoring systems substantially reduces the cost and the time required for test development and facilitates adaptation to different languages and cultural contexts. Although these approaches require further validation, the results included in this review indicate that their performance is comparable to that of traditional tests based on recorded speech stimuli, opening promising perspectives for the global dissemination of SIN assessment tools.

In recent years, artificial intelligence has increasingly been applied in healthcare, including otolaryngology and audiology, with a growing number of studies exploring its potential in auditory assessment [23,24,25,26,27]. Notably, one of the most innovative developments highlighted in this review is the application of machine learning (ML) methods to data derived from speech-in-noise (SIN) tests [15]. Unlike conventional approaches that rely primarily on single outcome measures, such as speech reception thresholds, this study demonstrated that multivariate ML models can integrate multiple dimensions of auditory performance, including demographic and behavioral features such as age, response accuracy, and reaction time. This multivariate approach not only improves the identification of hearing loss, achieving high levels of accuracy, sensitivity, and specificity, but also enables a more detailed characterization of auditory function. Furthermore, the incorporation of explainability techniques provides valuable insights into the relative contribution of individual features, enhancing the interpretability of model predictions and supporting clinical decision-making. Overall, this represents a significant paradigm shift from traditional unidimensional assessment methods toward a more comprehensive, data-driven, and potentially personalized evaluation of hearing abilities, with important implications for large-scale screening, early detection, and targeted intervention strategies.

Overall, the studies included in this scoping review support the idea that SIN tests can provide complementary and, in some cases, more sensitive information than traditional audiometric measures. Notably, the consistent ability to reliably differentiate between normal hearing listeners and those with hearing loss, observed across all included studies, reinforces the role of SIN testing as a key tool for the early identification of hearing loss and timely intervention. This aspect is of particular relevance considering the growing evidence linking untreated hearing loss to long-term cognitive and functional consequences.

An important aspect, considering the reviewed studies, concerns the reliability and reproducibility of SIN-based assessment tools. Several studies reported good agreement with reference tests and acceptable test–retest reliability, particularly for digit-based paradigms and automated implementations [9,18]. However, variability across testing conditions, devices, and listening environments remains a relevant issue, especially for remote and self-administered applications. Future developments should therefore focus on improving the standardization of test procedures, calibration across different hardware platforms, and validation in diverse populations and real-world settings. Additionally, further work is needed to establish normative data and clinically meaningful thresholds to enhance the interpretability and comparability of results across different SIN tools.

Another important limitation of SIN testing relates to its limited ability to distinguish between different types of hearing loss, such as conductive and sensorineural hearing loss. SIN tests primarily assess functional speech perception in noise and rely on global measures of auditory performance rather than frequency-specific thresholds. As a result, different pathophysiological mechanisms affecting audibility and signal processing may lead to similar performance outcomes in SIN tasks [5,7]. Although certain paradigms, such as antiphasic presentations, have shown increased sensitivity to specific configurations (e.g., unilateral or conductive hearing loss), these approaches do not provide sufficient specificity to replace conventional audiological assessments for differential diagnosis [16]. Therefore, SIN testing should be considered a complementary tool, useful for screening and functional evaluation, but not a standalone method for identifying the underlying type of hearing impairment.

Limitations. Firstly, the heterogeneity of the paradigms employed, the populations studied, and the outcome measures reported limit a direct comparison across tools. Furthermore, several of the proposed tools are still in the validation phase and therefore require further investigation to determine their long-term clinical impact and integration into standard diagnostic pathways.

## 5. Conclusions

In conclusion, this scoping review demonstrates that the field of speech-in-noise assessment has gone through a rapid and substantial evolution over the past decade. The emerging SIN tools reviewed in this manuscript show considerable potential to improve the identification and characterization of hearing loss, supporting more functional, accessible, and sensitive assessment approaches compared with conventional audiometry. Future research should focus on clarifying how these tools can be effectively integrated into clinical practice and hearing screening procedures, with the goal of promoting earlier diagnosis and timely intervention.

## Figures and Tables

**Table 1:** Characteristics of the included studies.

Author (Year)	Speech-in-Noise Tool	Population	Study Design	Setting
Ceccato et al. (2021) [13]	Antiphasic DIN (French)	167 (39.5% NH)	Validation (large-scale)	Smartphone
De Sousa et al. (2022) [14]	Diotic + antiphasic DIN	489 (59.9% NH)	Diagnostic/triage	Remote
Lenatti et al. (2022) [15]	SIN screening + ML	215 ears (53.9% NH)	Classification study	Clinical/Remote
Polspoel et al. (2023) [16]	Antiphasic DIN	36 (100% NH and simulated CHL)	Experimental (simulated CHL)	Laboratory
Schmid et al. (2023) [10]	Matrix SIN (BPACE)	40 (50% NH, 50% CI users)	Experimental comparison	Clinical
Banks et al. (2024) [17]	Digital Speech Hearing Screener	50 (54% NH)	Observational	App-based
Viola et al. (2024) [18]	DIN (Italian)	107 (42% NH)	Development and validation	App-based
Fatehifar et al. (2025) [9]	DIN (TTS + ASR)	31 (67.7% NH)	Proof-of-concept	Remote
Polspoel et al. (2025) [19]	Aladdin DIN	48 (58.3% NH)	Validation study	Remote

Abbreviations: ASR: automatic speech recognition; Aladdin: automatic language-independent development of the digits-in-noise test; BPACE: Bayesian PAtient-Centered; CI, cochlear implant; CHL: conductive hearing loss; DIN: digits-in-noise; ML: machine learning; NH, normal hearing; SIN, speech-in-noise; TTS: text-to-speech.

**Table 2:** Type of speech-in-noise tools and methodological innovations.

Author (Year)	Speech Material	Presentation Mode	Key Innovation
Ceccato et al. (2021) [13]	Digit triplets	Antiphasic	App-based large-scale screening
De Sousa et al. (2022) [14]	Digit triplets	Diotic + antiphasic	Classification and triage of hearing loss type
Lenatti et al. (2022) [15]	Speech-in-noise stimuli	Diotic	Machine learning with explainability techniques
Polspoel et al. (2023) [16]	Digit triplets	Antiphasic	Evaluation of sensitivity to simulated CHL
Schmid et al. (2023) [10]	Matrix sentences	Diotic	Bayesian adaptive procedure, reduced listening effort
Banks et al. (2024) [17]	Nonsense words	Monaural	Rapid digital screening (<3 min)
Viola et al. (2024) [18]	Digit triplets	Monaural (ear-specific)	Optimized adaptive algorithm
Fatehifar et al. (2025) [9]	Digit triplets (synthetic speech)	Diotic	Fully automated DIN using TTS and ASR
Polspoel et al. (2025) [19]	Digit triplets (TTS-generated)	Diotic	Automatic, language-independent DIN development

Abbreviations: ASR: automatic speech recognition; CHL: conductive hearing loss; DIN: digits-in-noise; TTS: text-to-speech.

**Table 3:** Outcome measures and clinical relevance.

Author (Year)	Main Outcome Measures	Key Quantitative Findings	Clinical Relevance
Ceccato et al. (2021) [13]	ROC curves, Z-scores	Sensitivity: 0.96; specificity: 0.93 for PTA above 20 dB	Large-scale screening
De Sousa et al. (2022) [14]	SRT, BILD, classification accuracy	75–79% correct classification of hearing status	Clinical triage
Lenatti et al. (2022) [15]	ML accuracy, feature importance	Accuracy: 0.85; sensitivity: 0.86; specificity: 0.85	AI screening
Polspoel et al. (2023) [16]	Effect of presentation level on SRT	Demonstrated SRT stability above 60 dB SPL	Paradigm validation
Schmid et al. (2023) [10]	SRT, psychometric slope, listening effort	Maintains diagnostic accuracy while reducing listening effort and test duration	Functional assessment
Banks et al. (2024) [17]	Classification accuracy vs. PTA/SRT	Accuracy 81.6–83.7% depending on PTA threshold	Hearing screening
Viola et al. (2024) [18]	Ear-specific SRT	Reliable estimation of SRT for each ear	Screening/assessment
Fatehifar et al. (2025) [9]	SRT, agreement, reliability	Agreement −1.3 ± 4.9 dB with reference test; reliability −1.0 ± 5.7 dB	Remote assessment
Polspoel et al. (2025) [19]	SRT, sensitivity, specificity	Sensitivity 100%; specificity 84%	Population screening

Abbreviations: BILD: binaural intelligibility level difference; ML: machine learning; PTA, pure-tone average; ROC: receiver operating characteristic; SRT: speech reception threshold; SPL, sound Pressure level.

## Data Availability

No new data were created or analyzed in this study. Data sharing is not applicable to this article.

## References

[1] World Health Organization (2021). 1 in 4 People Projected to Have Hearing Problems by 2050. https://www.who.int/news/item/02-03-2021-who-1-in-4-people-projected-to-have-hearing-problems-by-2050.

[2] Readman M.R., Littlejohn J., Dodd I., Rhodes S., Wareing L., Polden M., Plack C.J., Giebel C. (2025). Hearing loss as a risk factor for dementia: A systematic review and meta-analysis from a global perspective. Aging Ment. Health.

[3] Loughrey D.G., Kelly M.E., Kelley G.A., Brennan S., Lawlor B.A. (2018). Association of age-related hearing loss with cognitive function, cognitive impairment, and dementia: A systematic review and meta-analysis. JAMA Otolaryngol. Head Neck Surg..

[4] Wei J., Hu Y., Zhang L., Hao Q., Yang R., Lu H., Zhang X., Chandrasekar E.K. (2017). Hearing impairment, mild cognitive impairment, and dementia: A meta-analysis of cohort studies. Dement. Geriatr. Cogn. Disord. Extra.

[5] Pienkowski M. (2017). On the etiology of listening difficulties in noise despite clinically normal audiograms. Ear Hear..

[6] American Speech-Language-Hearing Association (2005). Guidelines for Manual Pure-Tone Threshold Audiometry.

[7] Ruggles D., Bharadwaj H., Shinn-Cunningham B.G. (2011). Normal hearing is not enough to guarantee robust encoding of suprathreshold features important in everyday communication. Proc. Natl. Acad. Sci. USA.

[8] Moore D.R., Edmondson-Jones M., Dawes P., Fortnum H., McCormack A., Pierzycki R.H., Munro K.J. (2014). Relation between speech-in-noise threshold, hearing loss and cognition from 40–69 years of age. PLoS ONE.

[9] Fatehifar M., Munro K.J., Stone M.A., Wong D., Cootes T., Schlittenlacher J. (2025). Digits-in-noise hearing test using text-to-speech and automatic speech recognition: Proof-of-concept study. Trends Hear..

[10] Schmid C., Wimmer W., Kompis M. (2023). BPACE: A Bayesian, patient-centered procedure for matrix speech tests in noise. Trends Hear..

[11] Herbert N., Keller M., Derleth P., Kühnel V., Strelcyk O. (2023). Optimised adaptive procedures and analysis methods for conducting speech-in-noise tests. Int. J. Audiol..

[12] Tricco A.C., Lillie E., Zarin W., O’Brien K.K., Colquhoun H., Levac D., Moher D., Peters M.D.J., Horsley T., Weeks L. (2018). PRISMA Extension for Scoping Reviews (PRISMA-ScR): Checklist and Explanation. Ann. Intern. Med..

[13] Ceccato J.C., Duran M.J., Swanepoel W., Smits C., De Sousa K.C., Gledhill L., Venail F., Puel J.L. (2021). French version of the antiphasic digits-in-noise test for smartphone hearing screening. Front. Public Health.

[14] De Sousa K.C., Smits C., Moore D.R., Myburgh H.C., Swanepoel W. (2022). Diotic and antiphasic digits-in-noise testing as a hearing screening and triage tool to classify type of hearing loss. Ear Hear..

[15] Lenatti M., Moreno-Sánchez P.A., Polo E.M., Mollura M., Barbieri R., Paglialonga A. (2022). Evaluation of machine learning algorithms and explainability techniques to detect hearing loss from a speech-in-noise screening test. Am. J. Audiol..

[16] Polspoel S., Moore D.R., Swanepoel W., Kramer S.E., Smits C. (2023). Sensitivity of the antiphasic digits-in-noise test to simulated unilateral and bilateral conductive hearing loss. Int. J. Audiol..

[17] Banks R., Greene B.R., Morrow I., Ciesla M., Woolever D., Tobyne S., Gomes-Osman J., Jannati A., Showalter J., Bates D. (2024). Digital speech hearing screening using a quick novel mobile hearing impairment assessment: An observational correlation study. Sci. Rep..

[18] Viola P., Astorina A., Mancuso A., Presti G., Scarpa A., Baruffini C., Chiarella G. (2024). Development and validation of the Italian digit-in-noise test. Acta Otorhinolaryngol. Ital..

[19] Polspoel S., Moore D.R., Swanepoel W., Kramer S.E., Smits C. (2025). Automatic development of speech-in-noise hearing tests using machine learning. Sci. Rep..

[20] Dingemanse G., Goedegebure A. (2019). Efficient adaptive speech reception threshold measurements using stochastic approximation algorithms. Trends Hear..

[21] Kollmeier B., Warzybok A., Hochmuth S., Zokoll M.A., Uslar V., Brand T., Wagener K.C. (2015). The multilingual matrix test: Principles, applications, and comparison across languages: A review. Int. J. Audiol..

[22] Schimmel C., Cormier K., Manchaiah V., Swanepoel W., Sharma A. (2024). Digits-in-noise test as an assessment tool for hearing loss and hearing aids. Audiol. Res..

[23] Migliorelli A., Manuelli M., Ciorba A., Stomeo F., Pelucchi S., Bianchini C. (2024). Role of artificial intelligence in human papillomavirus status prediction for oropharyngeal cancer: A scoping review. Cancers.

[24] Amanian A., Heffernan A., Ishii M., Creighton F.X., Thamboo A. (2023). The evolution and application of artificial intelligence in rhinology: A state-of-the-art review. Otolaryngol. Head Neck Surg..

[25] Frosolini A., Franz L., Caragli V., Genovese E., de Filippis C., Marioni G. (2024). Artificial intelligence in audiology: A scoping review of current applications and future directions. Sensors.

[26] Healy E.W., Johnson E.M., Pandey A., Wang D. (2023). Progress made in the efficacy and viability of deep-learning-based noise reduction. J. Acoust. Soc. Am..

[27] Ashkanichenarlogh V., Folkeard P., Scollie S., Kühnel V., Parsa V. (2025). Objective evaluation of a deep learning-based noise reduction algorithm for hearing aids under diverse fitting and listening conditions. Trends Hear..

